# Combined Pediatric T-condylar Humeral Fracture and Monteggia Fracture-Dislocation in the Upper Extremity: A Case Report

**DOI:** 10.7759/cureus.81866

**Published:** 2025-04-08

**Authors:** Ryo Yakushiji, Takeshi Ogawa, Akira Ikumi, Kenji Kobayashi, Yuichi Yoshii

**Affiliations:** 1 Department of Orthopedic Surgery, National Hospital Organization, Mito Medical Center, Ibaraki, JPN; 2 Department of Orthopedic Surgery, University of Tsukuba, Tsukuba, JPN; 3 Department of Orthopedic Surgery, Tokyo Medical University Ibaraki Medical Center, Ibaraki, JPN

**Keywords:** humeral condyle fracture, kirschner wire fixation, monteggia fracture, pediatric orthopedics, t-fracture

## Abstract

Monteggia fractures are characterized by a fracture of the ulna associated with radial head dislocation. T-condylar fractures of the humerus are intra-articular injuries that disrupt the distal humeral epiphysis. This report describes a rare case of a pediatric patient with an ipsilateral T-condylar humeral fracture and a Monteggia fracture-dislocation. A 10-year-old girl presented with elbow pain and deformity after falling while playing basketball. No open wounds or neurological deficits were observed, and the radial artery was palpable. The radiograph revealed a pediatric T-condylar humeral fracture and a Bado classification type I Monteggia fracture-dislocation. Surgery was performed on the day of injury, and the ulnar shaft fracture was repaired with a titanium elastic nail. Subsequently, the humeral condyle was stabilized with Kirschner wire fixation. Bone union was confirmed at two months postoperatively, and the nails and wires were removed at six months postoperatively. Two years post-surgery, the patient had full elbow range of motion with no growth disturbances, deformities, or pain. This rare upper extremity fracture-dislocation case illustrates the need for prompt surgery and long-term follow-up to monitor growth and function.

## Introduction

The incidence of ipsilateral supracondylar humeral fractures combined with forearm fractures, including in adults, is reported to be 1-13% [1]. To our knowledge, no cases of pediatric T-condylar humeral fractures combined with Monteggia fracture-dislocation have been reported in the literature. Pediatric T-condylar fractures of the humerus are intra-articular injuries that disrupt the distal humeral epiphysis, posing a risk to joint congruity and long-term elbow function. These pediatric T-condylar humeral fractures are rare, accounting for approximately 2% of elbow fractures in children, and most of the literature on these fractures is limited to case reports and case series [2-6]. Additionally, Monteggia fracture-dislocations are uncommon injuries, accounting for around 2% of all elbow fractures in children [7]. Several case reports describe children with Monteggia fracture-dislocation combined with other ipsilateral upper extremity injuries. However, most of these injuries affected the forearm [8]. We experienced an extremely rare case of an ipsilateral humeral condylar fracture combined with a Monteggia fracture-dislocation. The simultaneous occurrence of these injuries in the same limb poses unique diagnostic and therapeutic challenges. This case report describes the clinical course of this rare injury and discusses its pathogenesis.

## Case presentation

Patient

The patient is a 10-year-old girl with no medical history.

History

The patient fell during contact with other players while playing basketball, sustaining an injury. She experienced pain and swelling in her left upper limb and was referred to our hospital for surgery after being sent to a local medical institution for emergency treatment.

**Figure 1:**
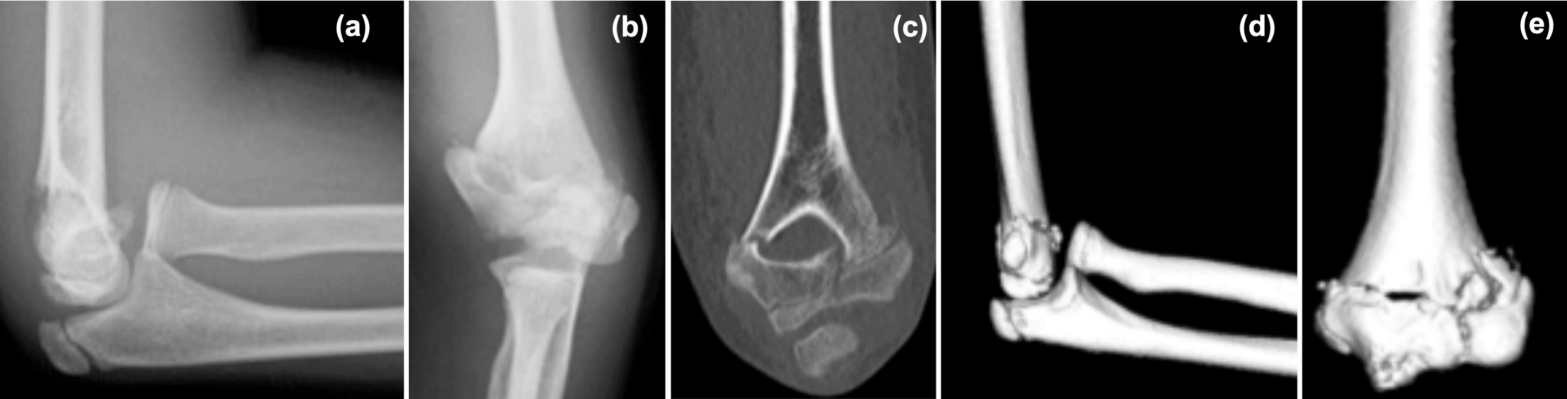
Elbow images on the day of injury (a) X-ray lateral view. (b) X-ray anteroposterior view. (c-e) Computed tomography images suggested a fracture line in the humeral condyle, consistent with a pediatric T-condylar fracture.

**Figure 2:**
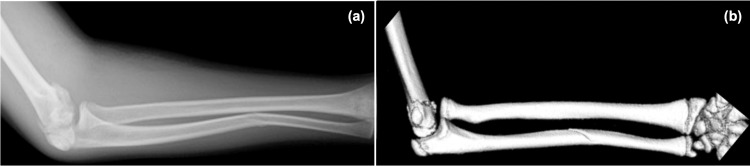
Forearm images on the day of injury (a) X-ray. (b) Computed tomography. Both showed an anterior dislocation of the radial head and an anterior convex to angulated deformity of the ulnar diaphysis.

At the time of the initial examination, no evidence of open wounds or limited finger motion was found, and the radial artery was well palpated. The patient's elbow joint was markedly swollen, and her forearm was curved ulnarly. No neurological symptoms, such as numbness, were observed. Plain X-ray and computed tomography of the elbow joint and forearm were performed. Imaging revealed a left humeral condylar fracture (AO Foundation/Orthopaedic Trauma Association (AO/OTA) classification 13C1, pediatric T-condylar fracture) [9] and Monteggia fracture-dislocation (Bado classification type I) [10] (Figure 1 and Figure 2).

Emergency surgery was performed due to significant swelling, deformity, and concerns about circulatory disturbance and compartment syndrome.

Surgical procedures

**Figure 3:**
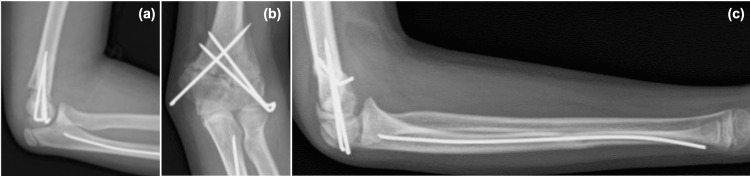
Images immediately after surgery (a, b) Pinning of the distal humerus by three Kirschner wires. (c) Insertion of a titanium elastic nail into the ulna.

First, a 2-mm-diameter titanium elastic nail was inserted from the distal ulna, and the ulnar shaft fracture was repaired and fixed internally. However, the radial head was not reduced; therefore, a skin incision was made on the lateral side of the elbow, and the humeroradial joint was checked using Kaplan's approach. After partially removing and releasing the annular ligament, the radial head was reduced. The stability of the humeral condyle was confirmed by palpation. The humeral condylar fracture was well aligned and stable, but the proximal fragment of the condyle remained unstable. Rotation and extension deformity were manually reduced, and three 2-mm-diameter Kirschner wires were inserted (two from the lateral side and one from the medial side) to achieve fixation (Figure 3). The lateral Kirschner wires were inserted percutaneously, and the medial Kirschner wire was fixed with a mini-open technique to prevent the ulnar nerve injury.

The patient was placed in a long arm cast above the elbow for four weeks, with the elbow in 90° flexion and the forearm in a neutral position. At four weeks postoperatively, the patient had the Kirschner wires removed and was switched to a long arm posterior splint and allowed to perform active pronation and supination. The range of motion (ROM) at this point was 115° of elbow flexion, -30° of elbow extension, 70° of forearm pronation, and 90° of forearm supination.

**Figure 4:**
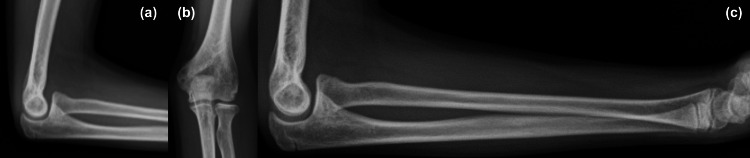
Images at the one-year postoperative follow-up (a-c) Complete bone union with no deformity.

Elastic nail removal was performed at five months postoperatively. At that time, ROM was 120° of elbow flexion, -30° of elbow extension, 80° of forearm pronation, and 90° of forearm supination, and the patient returned to the original athletic activities. At the one-year postoperative follow-up, ROM was 135° for elbow flexion, +10° for elbow extension, 80° for forearm pronation, and 90° for forearm supination, with no ROM limitation or growth disturbance (Figure 4).

At the two-year postoperative follow-up, the ROM was 140° for elbow flexion, +10° for elbow extension, 80° for forearm pronation, and 90° for forearm supination. The patient had full elbow ROM, with no activity limitations, growth disturbance, deformity, or pain.

## Discussion

The mechanism of injury in pediatric T-condylar humeral fractures [9] involves a direct wedge effect of the olecranon's articular surface at the distal end of the humerus. The sharp edge of the semilunar notch, or coronoid process, then acts as a wedge, breaking the trochlea and splitting the condyles. Either flexion or extension injuries cause this condition. The flexion mechanism occurs directly below the posterior aspect of the elbow. Usually, the child falls directly on the flexed elbow. Conversely, the extension mechanism involves a wedge effect by the coronoid portion of the semilunar notch [3,7]. In flexion injuries, condylar fragments are anterior to the shaft, while in extension injuries, they are posterior [1]. These mechanisms are similar in adults [11]. However, in adults, contrecoup impaction towards the lower end of the humeral shaft is suggested to result in the separation of the medial and lateral columns [12].　

The mechanism of injury in Monteggia fracture-dislocation, according to the Bado classification type I [10], has been reported using three different theories. In the direct external force theory, the ulnar diaphysis is fractured by a direct external force from the dorsal side, pushing the diaphysis forward and dislocating the radial head due to forearm rotation [8]. In the hyperpronation theory, the ulna pushes the radial head forward and causes dislocations owing to forearm pronation [13]. According to the hyperextension theory, the ulnar diaphysis fractures after the dislocation of the radial head are due to elbow hyperextension [14].

**Figure 5:**
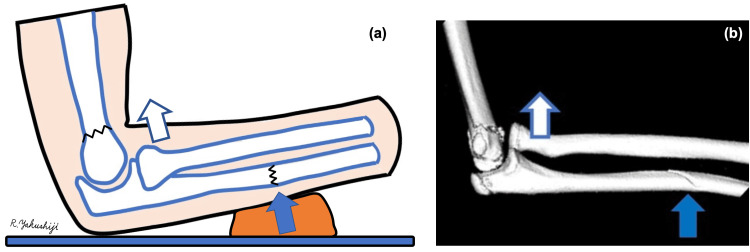
Pictures reproduced by the author demonstrating the mechanism of Bado classification type I Monteggia fracture-dislocation. (a) Schema drawn by the first author. (b). 3D-CT of a part of the presented case. The forearm was pressed against the shoe, which then pushed on the ulnar shaft (solid arrow). Subsequently, the radial head dislocated anteriorly (open arrow). 3D-CT: three-dimensional computed tomography

In this case, we hypothesized that the ulna was deformed anteriorly by a direct external force (Figure 5, solid arrow) and that the radial head was dislocated anteriorly (Figure 5, open arrow), resulting in the Monteggia fracture-dislocation.

**Figure 6:**
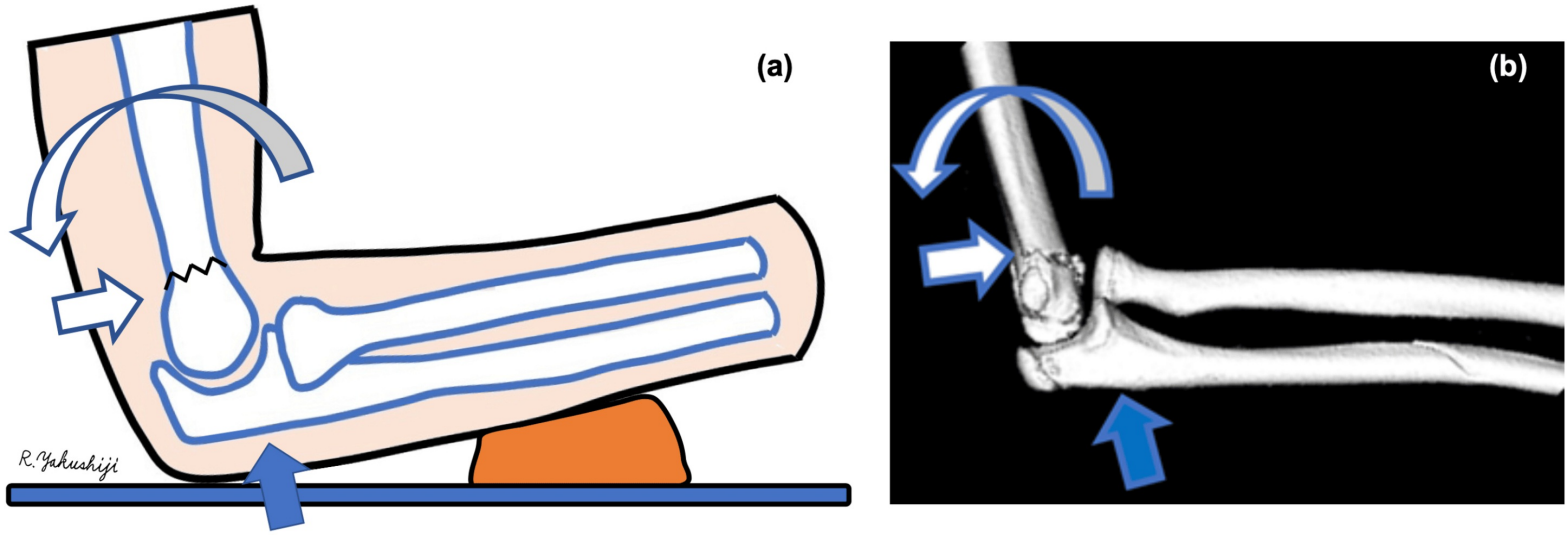
Pictures reproduced by the author demonstrating the mechanism of a pediatric T-condylar fracture (a) Schema drawn by the first author. (b) 3D-CT of a part of the presented case. The olecranon impacted the ground and exerted longitudinal pressure on the humerus (solid arrow). The humerus was dislocated in the extension direction (open arrows). 3D-CT: three-dimensional computed tomography

As for the humeral condylar fracture, displacement occurred in the direction of extension. With the forearm on the shoe, the elbow was placed on the ground (Figure 6, solid arrow), the ulna pushed the humeral pulley away, and the humerus was subjected to an intervening external force in the direction of extension, resulting in the distal humerus being displaced towards extension (Figure 6, open arrows) and the patient sustaining an extension humeral condylar fracture.

Whether the humerus or the forearm should be fixed first remains controversial. Since this problem affects the approach to be used, the posterior approach is used for pediatric T-condylar humeral fractures. In this approach, the triceps is spared and enhanced with an olecranon osteotomy when necessary [5]. No established opinion exists regarding the complications of humeral condylar fractures and Monteggia fracture-dislocations in children, necessitating the careful consideration of the treatment plan for each case. In this case, the ulna was fixed with an elastic nail, and the radial head could not be reduced; therefore, we attempted to reduce the radial head using Kaplan's approach. The annular ligament was interposed in the joint, preventing the reduction of the radial head. The same skin incision provided direct access to the intra-articular fracture distal to the humerus, which was useful in both cases. Although the posterior approach is often recommended for humeral condylar fractures, it does not allow for the reduction of radial head dislocations. It is effective to first perform ulnar repair and fixation to determine whether a closed reduction of the radial head dislocation is possible before treating a humeral condylar fracture. When the radial head dislocation can be reduced by a closed method and the humeral condylar fracture is severely comminuted, the posterior approach is chosen for direct repair [3].

## Conclusions

We report a case of combined pediatric T-condylar humeral fracture and Monteggia fracture-dislocation. The fracture mechanism involved a chain reaction of direct external force on the ulnar diaphysis and an intervening external force in the direction of humeral extension. Kaplan's approach was useful in this case as it allowed both the open reduction of the radial head anterior dislocation and the direct reduction of the humeral condylar fracture fragment through the same skin incision.
